# MTBP regulates cell survival and therapeutic sensitivity in TP53 wildtype glioblastomas

**DOI:** 10.7150/thno.35747

**Published:** 2019-08-14

**Authors:** Yifu Song, Li Zhang, Yang Jiang, Tianhao Hu, Di Zhang, Qiao Qiao, Run Wang, Minghao Wang, Sheng Han

**Affiliations:** 1 Department of Neurosurgery, The First Hospital of China Medical University, Shenyang 110001, China; 2 Department of Pathology, China Medical University, Shenyang 110001, China; 3 Department of Neurosurgery, Shanghai First People's Hospital of Shanghai Jiao Tong University School of Medicine, Shanghai 200080, China; 4 Department of Radiotherapy, The First Hospital of China Medical University, Shenyang 110001, China

**Keywords:** Glioma, MTBP, MDM2, p53, apoptosis

## Abstract

**Background**: Glioblastoma (GBM) is highly proliferative and resistant to radio-chemotherapy. Loss of tumor suppressor gene TP53 function frequently occurs at protein level in GBMs. This inhibition is often mediated by other components within the p53 signaling axis, including MDM2, whose binding protein (MTBP) plays an important role in the regulation of MDM2 and p53 activity. We investigated the role of MTBP in the biology of TP53-wildtype (TP53wt) GBMs.

**Methods**: MTBP expression was examined in TCGA and REMBRANDT datasets. MTBP was silenced or overexpressed in TP53wt GBM cells and glioma stem cells (GSCs). The effects on cell viability, apoptosis, and clonogenicity were assessed. The transcriptional regulation of MTBP was investigated.

**Results**: Upregulation of MTBP was correlated with the Classical molecular subtype, and it predicted poor survival. In TP53wt GBM cells, the protein levels of MTBP were positively associated with those of MDM2 but negatively correlated with those of p53. MTBP knockdown promoted apoptosis and inhibited clonogenicity, while overexpression of this protein enhanced tumorigenicity *in vitro* and *in vivo*. The pro-survival effect of MTBP depended on the activity of MDM2 and p53. MTBP was transcriptionally regulated by c-myc, thereby forming a positive regulatory loop. Finally, MTBP silencing increased the sensitivity of TP53wt GSCs to radiation and TMZ treatment *in vitro* and* in vivo*.

**Conclusion**: MTBP regulates the cell survival and treatment sensitivity of TP53wt GBMs through MDM2-dependent post-translational modification of p53. MTBP-targeting treatments are potentially useful in increasing patients' survival.

## Introduction

Glioblastomas (GBMs) are the most common primary malignant brain tumors and are intrinsically insensitive to currently available radio-chemotherapy treatments 1 due to special cellular and molecular features. GBMs are composed of heterogeneous cell populations that include glioma stem cells (GSCs) with tumor initiation and self-renewal properties. GSCs are considered to be responsible for therapeutic resistance and tumor recurrence 2. However, the molecular basis that regulates the unique properties of these cells is largely unknown.

The tumor suppressor gene TP53 (p53) plays an important role in protecting cells against DNA damage and stress. Studies have shown that the activation of p53 transcriptional functions by carcinogens results in growth arrest and apoptosis 3. Malignant tumors often involve the mutation of the TP53 gene 4, and disruption of p53 functions ultimately gives rise to an oncogenic phenotype of neural stem cells 5. However, in the case of GBMs, the tumor usually harbors a wildtype TP53 (TP53wt) gene. Therefore, the augmented proliferation and resistance to cytotoxic treatment in TP53wt GBMs has been attributed to the loss of p53 functions by post-translational modification 3. Although p53 can be produced constitutively, it is negatively regulated by the mouse double minute 2 (MDM2) protein that binds to the transcription activation domain of p53, thereby inhibiting acetylation, stimulating nuclear export, and most importantly, promoting proteasomal degradation via MDM2's E3 ubiquitin ligase activity 6. The tumor-promoting effect of MDM2 in GBMs has been reported in prior publications 7.

The MDM2 binding protein (MTBP) is another important component of the p53 signaling axis. The MTBP gene is located on chromosome 8q24.12, and MTBP was first identified as a protein that directly interacts with MDM2 8. Subsequently, MTBP has been found to be upregulated in various cancer cells, including lung cancer, breast ductal carcinoma, cervical carcinoma, and colorectal carcinoma 9. By regulating the E3 ubiquitin ligase activity of MDM2, MTBP promotes both MDM2 stabilization and p53 degradation 6. Therefore, the upregulation of MTBP results in elevated MDM2 level and reduced p53 activity. Although MTBP contributes significantly to MDM2-dependent p53 homeostasis, its role in GBMs has not yet been explored. In addition, MTBP could be a potential candidate for regulating p53 as an inhibitor via the MTBP-MDM2-p53 pathway in GBMs. This study investigates the effect of MTBP on cell survival and treatment sensitivity in TP53wt GBMs.

## Materials and Methods

### Ethics

The protocols set by the institutional review board of The First Hospital of China Medical University were strictly observed in this study. All donors provided their written consent that their tumor tissue and clinical data be used for future research. Animal work was conducted in accordance with the China Medical University Animal Ethics Committee guidelines and approved by the Institutional Review Board of The First Hospital of China Medical University (Approval number 2017001M).

### Cell culture and GSCs isolation

The human TP53mut LN229 GBM cell line 10 was obtained from iCell Bioscience Inc. (Shanghai, China) in December 2017, while the TP53wt U87 cell line 10 was purchased from the Chinese Academy of Sciences cell bank (Shanghai, China) in September 2016. Cells were cultured in Dulbecco's modified Eagle medium (DMEM; HyClone, Logan, UT, USA), supplemented with 10% fetal bovine serum (FBS; Gibco, Carlsbad, CA, USA) and 1% penicillin/streptomycin (Gibco) at 37ºC with 5% CO_2_. Mycoplasma tests were performed on the cultured cells on a monthly basis using the MycoAlert Mycoplasma Detection Kit (Lonza, Switzerland). LN229 and U87 were authenticated using STR DNA profiling by Genecreate Biotech (Wuhan, China) in May 2018. The cells used were limited to less than 12 passages.

Patient-derived primary glioma cells (GC-1710, GF-1712, GS-1802, and GW-1806) were isolated, and neurosphere cultures were performed as previously described 11. Briefly, freshly resected glioma specimens were dissociated into single cells and grown in serum-free DMEM/F12 with 2% B27, 20 ng/mL rh-bFGF, and 20 ng/mL rh-EGF (Gibco). Free-floating neurospheres were collected and routinely cultured in the above-mentioned neurosphere medium. The TP53, IDH1/2, 1p/19q, TERT, and MGMT promoter methylation status were examined using next-generation sequencing and pyrosequencing by GenomiCare Biotechnology (Shanghai, China). GSC isolation from U87 and LN229 cells was performed as detailed in previous studies 11, 12, and CD15^+^ GSCs were isolated using magnetic cell sorting (MACS) with CD15 microbeads (Miltenyi Biotec, 130-046-601). The cancer stem cell nature of the isolated GSCs was confirmed by functional assays of self-renewal, multi-lineage differentiation, and *in vivo* tumor formation. The expression of stem cell marker CD15 13, 14 was evaluated by flow cytometry.

### Real-time PCR analysis

Total RNA was isolated using Trizol reagent (Invitrogen, Eugene, OR, USA). The first-strand cDNA was synthesized with Prime-Script RT Master Mix (TaKaRa, Kyoto, Japan), followed by qPCR detection using the SYBR Green Master Mix (TaKaRa). Each sample was run four times using β-actin as internal control. The following primers were used: MTBP F: 5′-TCCTGTAGTTTCGTCAGATCCT-3′ and R: 5′-CCGTTTCAATCGGGATACTTCA-3′; MDM2 F: 5′-TGTAAGTGAACATTCAGGTG-3′ and R: 5′-TTCCAATAGTCAGCTAAGGA-3′; and p53 F: 5′-CCGGCGCACAGAGGAAGAGA-3′ and R: 5′-TGGGGAGAGG AGCTGGTGTTGT-3′.

### Western blotting analysis

Western blotting analysis was performed as previously described 12. Briefly, total proteins that were extracted using a Total Cell Protein Extraction Kit (KeyGen Biotechnology, Nanjing, China) were divided into equal amounts and electrophoresed, transferred onto a nitrocellulose membrane, and blocked with 2% bovine serum albumin. Primary antibodies against MTBP (1:1000; ab115529, Abcam, Cambridge, UK), MDM2 (1:2000; ab16895), p53 (1:1000; ab131442), p21 (1:2000; ab109520), PUMA (1:2000; ab33906), active caspase3 (1:1000; ab2302), and c-myc (1:1000; ab56) were used to detect the expression of these proteins. After washing four times with TBST/0.1% Tween-20, the membranes were incubated with the corresponding secondary antibody. Bands were visualized using a chemiluminescence kit (Beyotime Biotechnology, Beijing, China) and quantified using the ImageJ software (National Institutes of Health, Bethesda, MD, USA).

### Immunohistochemistry (IHC)

IHC staining was performed, and the results were semi-quantified as previously reported 15. Paraffin-embedded sections were labeled with anti-MTBP (1:200; ab115529), anti-MDM2 (1:100; ab16895), or anti-p53 (1:100; ab131442) and were photographed with a light microscope (BX-51, Olympus, Tokyo, Japan).

### Lentivirus transduction

Independent shRNAs targeting MTBP or MYC, siRNAs targeting MDM2 or p53, and respective scrambled controls were obtained from GeneChem (Shanghai, China). The cDNAs of MTBP or MYC were cloned into lentivirus-based vectors (Gene-Chem) according to the manufacturer's instructions for overexpression. The lentivirus transduction was performed as described previously 11.

### Cell viability assays

Cells were plated in 96-well plates at 1 × 10^3^ cells/well and cultured for 0, 24, 48, 72, 96, and 120 h. The plates were examined using a cell viability assay kit (Promega, Madison, WI, USA) in accordance with the manufacturer's protocols, as described previously 16.

### EdU staining cell proliferation assays and colony formation assays

EdU staining was performed to visualize proliferating cells using a Click-iT^TM^ EdU Alexa Fluor® 488 Imaging kit (Invitrogen) according to the manufacturer's instructions. Cells were seeded into eight-well slides and cultured overnight. The following day, 10 µM EdU was added to the culture media and incubated for an hour. The cells were then fixed and permeabilized. Nuclei were counterstained with Hoechst 33342. Soft agar colony formation assays were performed as described in a previous study 16.

### Neurosphere formation assays

For limiting dilution neurosphere formation assays, GSCs were dissociated into single cells and seeded into 96-well plates at 50, 100, 500, or 1000 cells/well. Four days later, spheres with diameter > 50 μm were counted, and the data were analyzed. For radiation and temozolomide (TMZ) treatment assays, equal numbers of GSCs were pre-treated with 4 Gy radiation or 100 μM TMZ. One group of GSCs was used as control. The total number of spheres for treatment groups was recorded five days later and compared to the control cell sphere number that was set to 100%. Further details are reported in a previous study 11.

### Flow cytometry

To examine the cell cycle, cells were seeded into 6-well plates and cultured for 24 h. These cells were then trypsinized, washed with PBS, and stained with propidium iodide (PI, 75 μM) in the presence of NP-40. The cells were analyzed using a FACS Calibur flow cytometer (BD Biosciences, San Jose, CA, USA) and FlowJo V10 software (TreeStar, Ashland, OR, USA). The percentage of apoptotic cells was determined using an Annexin V Apoptosis Detection kit (BD Biosciences) and flow cytometry as per the manufacturer's protocols.

### Xenografts

The GSCs (1 × 10^5^ cells in 5 μL PBS) were stereotactically injected into the brains of 6-week-old female BALB/c nude mice as previously described 11. For radiation and TMZ treatment experiments, seven days after implantation, the mice were divided into treatment and control groups (n=5 per group). The radiation groups received radiation at 2 Gy for five consecutive days (MultiRad225, Faxitron, Tucson, AZ, USA). The TMZ treatment groups received 10 mg/kg/day TMZ. Mice were observed daily for signs of neurological symptoms and then sacrificed to evaluate tumor growth.

### Co-immunoprecipitation (co-IP)

Cells were resuspended in RIPA lysis buffer, followed by incubation with anti-MTBP (1:100; SC137201, Santa Cruz Biotechnology, Dallas, TX, USA) or anti-MDM2 (1:100; ab16895) overnight at 4°C. Then, the proteins were incubated with protein-A/G PLUS-Agarose beads (Millipore). The samples were further analyzed by western blot analysis and anti-IgG served as a negative control.

### Luciferase activity analysis

The MTBP promoter was cloned from U87 cells to encompass 1573-1073 base pairs upstream of the transcriptional initiation sites. Direct site mutagenesis of c-myc binding sites was generated using a QuikChange II XL Site-Directed Mutagenesis Kit (Agilent Technologies, Santa Clara, CA, USA) according to the manufacturer's instructions. Forty-eight hours later, the cells were lysed, and luciferase activity was detected using the Dual Luciferase Reporter Assay System (Promega, Fitchburg, WI, USA) as per the manufacturer's protocol 16.

### Chromatin immunoprecipitation (ChIP) assays

ChIP assays were performed using the EZ-ChIP ^TM^ Immunoprecipitation Kit (Millipore, Billerica, MA, USA) 16. The chromatin complexes were immuno-precipitated by anti-c-myc antibody (1:100; ab56), and the purified DNA samples were analyzed by qPCR. The primer pairs for the c-myc binding site in the MTBP promoter were f: 5′-CTCCACCAGATGCTTT-3′ and r: 5′-TTGTTGCCTGTCTGTG-3′. All reactions were performed in triplicate.

### Statistical analysis

Results are reported as the mean ± SEM of at least three independent experiments. The chi-square test, t test, and analysis of variance were used to examine the statistical significance among different groups. The Cancer Genome Atlas (TCGA) glioma dataset and Repository of Molecular Brain Neoplasia Data (REMBRANDT) database were accessed and processed via the GlioVis (http://gliovis.bioin-fo.cnio.es/) and GEPIA (http://gepia.cancer-pku.cn/) online platforms as previously reported 16-18. Survival differences were detected using the log-rank test and Kaplan-Meier analysis. Two-tailed P-values < 0.05 were regarded as significant. SPSS v.19.0 (SPSS Inc., Chicago, IL, USA) software was used for statistical analyses.

## Results

### Correlation between MTBP and p53 protein expression levels

**Figure 1:**
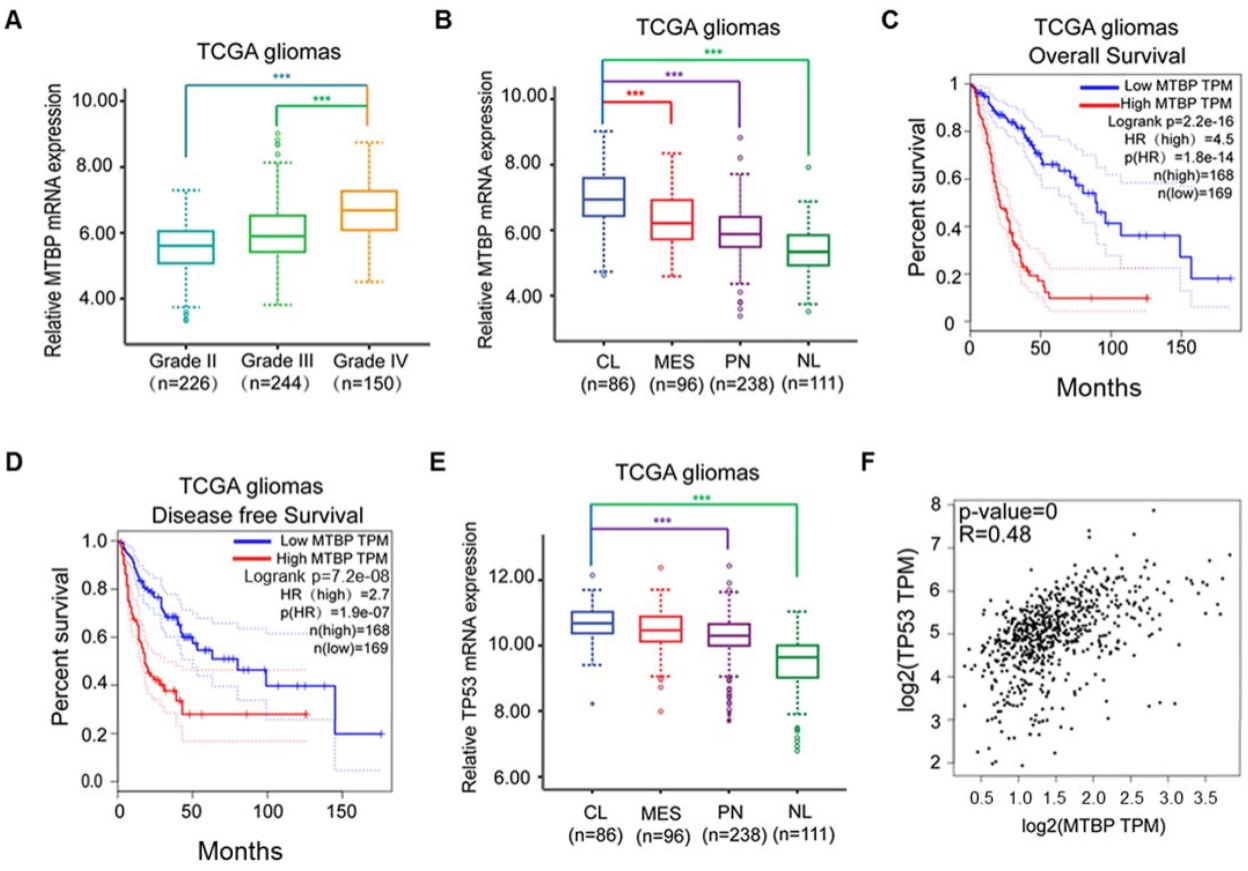
Influence of pathological and molecular glioma features on MTBP and TP53 expressions. A: Influence of TCGA glioma histopathologic grades on MTBP mRNA expression. B: Influence of TCGA glioma molecular subtypes on MTBP mRNA expression. C and D: Prognostic value of MTBP in TCGA gliomas. E: Influence of TCGA glioma molecular subtypes on the mRNA expression of TP53. F: Correlation between the MTBP mRNA and TP53 mRNA expressions. ***P < 0.001. Classical, CL; neural, NL; proneural, PN; and mesenchymal, MES.

MTBP mRNA expression in clinical gliomas was examined using TCGA and REMBRANDT datasets. In the case of TCGA gliomas, MTBP expression was associated with the histopathologic grade of glioma, with higher MTBP expression being observed in high-grade gliomas (Figure 1A; P<0.001). Moreover, compared with proneural, mesenchymal, and neural subtypes, MTBP expression was found to be significantly elevated in classical gliomas (Figure 1B; P<0.001). Increased MTBP expression also corresponded to poorer prognosis (Figure 1C and D; P<0.0001). Similar results were obtained for REMBRANDT gliomas (Figure S1A-C).

The assessment of TP53 mRNA expression in different molecular subtypes shows that it is highest in classical gliomas (Figure 1E and Figure S1D; p<0.01) and that the mRNA level of TP53 is positively correlated to that of MTBP (Figure 1F; P<0.001). Interestingly, although the TP53 gene is frequently mutated in other glioma subtypes, there is a distinct lack of TP53 mutations in the classical subtype 19. Nevertheless, higher TP53 mRNA expression is not conducive of patient recovery as it also corresponds to shorter survival time (Figures. S1E and F; P<0.0001). These results suggest that, in TP53wt gene-harboring classical gliomas, MTBP may affect the post-translational modification of p53, ultimately compromising its function.

**Figure 2:**
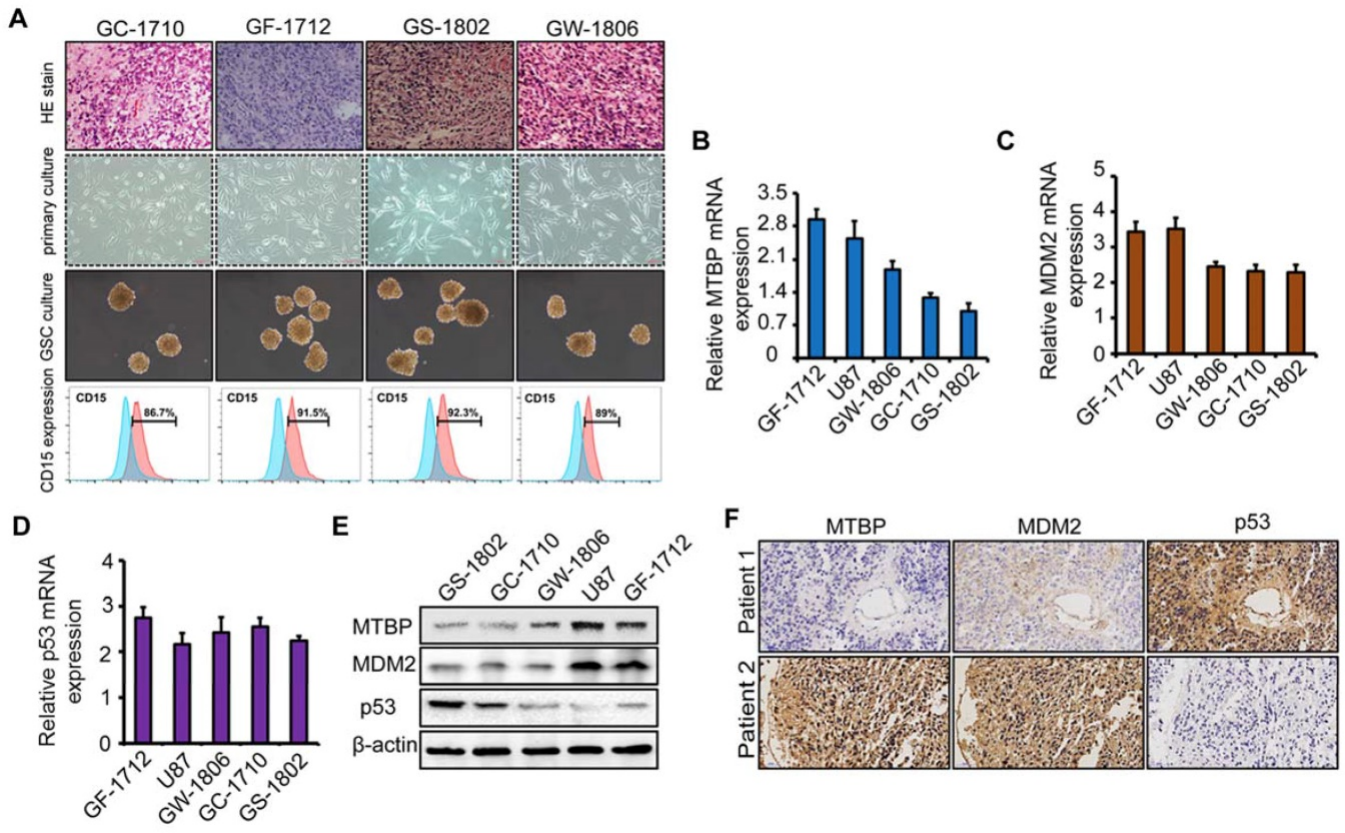
The association of MTBP, MDM2, and p53 expressions in TP53wt GBMs. A: HE stained images of the original patient tumors, patient-derived primary glioma and neurosphere cultures, and the expression of CD15 in GSCs of TP53wt GC-1710, GF-1712, GS-1802, and GW-1806. B-D: Real-time PCR analyses of the mRNA expressions of MTBP (B), MDM2 (C), and p53 (D). E: Western blotting analyses of MTBP, MDM2, and p53 protein levels. F: Staining of consecutive sections of clinical TP53wt GBM specimens for MTBP, MDM2, and p53 distribution assessment by IHC.

To explore the role of MTBP in TP53wt GBMs, patient-derived TP53wt primary glioma cells were established, and GSCs were isolated from neurosphere cultures (Figure 2A). Clinical data and molecular parameters (TP53, IDH1/2, 1p/19q, TERT, and MGMT promoter methylation status) of GC-1710, GF-1712, GS-1802, and GW-1806 are shown in Table S1. The GBMs were all IDH wildtype. The expression of the stem cell marker CD15 in isolated GSCs is presented in (Figure 2A), whereas the multi-lineage differentiation capacity of GSCs is shown in (Figure S1G). As shown in (Figures S1H and I), GC-1710, GF-1712, GS-1802, and GW-1806 highly expressed the classical markers EGRF and Nestin, while the expression of proneural marker Olig2 and mesenchymal marker YKL40 was low, indicating a classical subtype of these cells. mRNA (Figure 2B-D) and protein (Figure 2E) levels of MTBP, MDM2, and p53 in TP53wt glioma cells were examined, and it was found that, at protein level, MTBP expression is positively correlated with MDM2 expression and negatively correlated with p53 expression (Figure 2E). To characterize the intratumoral distribution of MTBP, MDM2, and p53, we assessed the expression of these proteins by IHC on serial paraffin-embedded sections of TP53wt GBM clinical specimens. Importantly, high MTBP expression areas coincided with MDM2 positivity and p53 negativity (Figure 2F). These findings suggest that MTBP negatively regulates the protein level of p53 in TP53wt GBMs.

### Effect of MTBP silencing on the growth of TP53wt GBM cells

**Figure 3:**
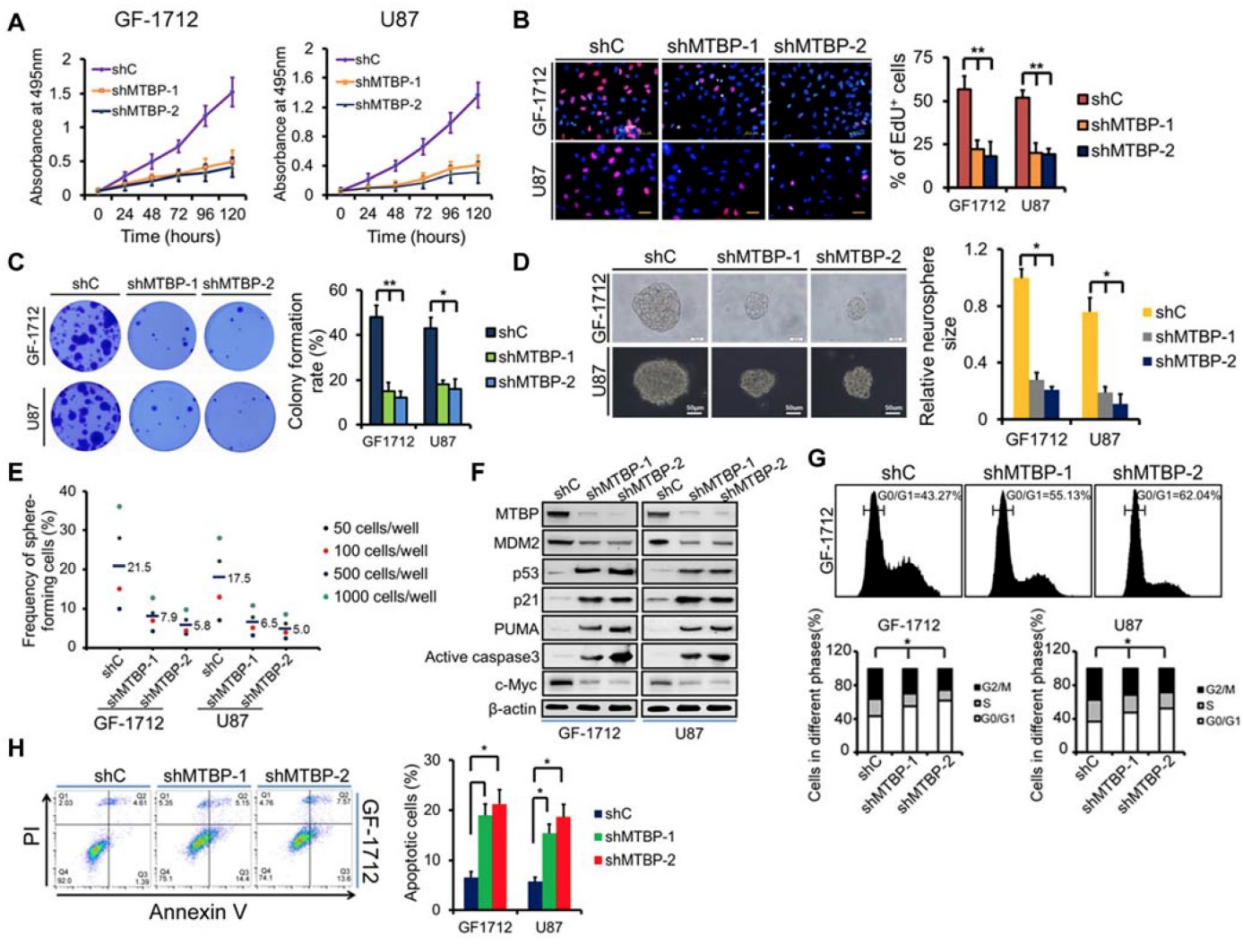
Effect of MTBP silencing on *in vitro* TP53wt GBM cell growth. A: Effect of MTBP knockdown by shRNA on cell viability in TP53wt GF-1712 and U87 cells. B: Representative images of EdU staining cell proliferation assay (left) and quantification of EdU-positive cells (right). Nuclei were counterstained with Hoechst 33342. Scale bar: 50 μm. C: Effect of MTBP silencing on TP53wt GBM cell colony formation. D: Representative images of GF-1712 and U87 neurospheres transduced with shRNA targeting MTBP, with ShC serving as a control (left). Quantification of relative neurosphere sizes of indicated GSCs (right). Scale bar: 20 μm (upper) and 50 μm (lower). E: Effect of MTBP silencing on the *in vitro* clonogenicity of GF-1712 and U87 GSCs. F: Western blotting analysis of MTBP, MDM2, p53, p21, PUMA, active caspase3 and c-myc protein levels in GF-1712 and U87 cells transfected with shRNAs targeting MTBP or a control shRNA. G: Effect of MTBP knockdown on G0/G1 in TP53wt GBM cells as determined by flow cytometry. H: Effect of MTBP silencing on the apoptosis of GF-1712 and U87 cells as demonstrated by Annexin V/PI staining and flow cytometry analyses. Results are presented as mean ± SEM of triplicate samples from three independent experiments. *P<0.05, **P < 0.01.

To examine the function of MTBP in TP53wt GBM cells, MTBP expression in GF-1712 and U87 cells that had high basal MTBP levels was silenced (Figure 2E) using two independent shRNAs. The results depicted in (Figure 3) show that knocking down MTBP markedly reduces the viability (Figure 3A; P<0.05), proliferation (Figure 3B; P<0.01), and colony formation (Figure 3C; P<0.05) of TP53wt GBM cells.

Moreover, shMTBP-infected GSCs show significantly decreased sphere-forming capacity (Figure 3D and E; P<0.05). These findings indicate that MTBP is essential to the growth of TP53wt GBM cells *in vitro*.

Western blotting analysis shows that MTBP silencing decreases MDM2 and c-myc protein levels while increasing the pro-apoptotic protein levels of p53, p21, PUMA, and active caspase3 (Figure 3F). Cell cycle analysis reveals that MTBP knockdown results in G0/G1 arrest of GF-1712 and U87 cells (Figure 3G; P<0.05). Moreover, shMTBP-infected cells show a substantial accumulation of apoptotic cells as judged by Annexin V/PI staining (Figure 3H; P<0.05). Therefore, MTBP silencing may induce p53-mediated apoptosis in TP53wt GBM cells.

### Effect of MTBP overexpression on the growth of TP53wt GBM cells

**Figure 4:**
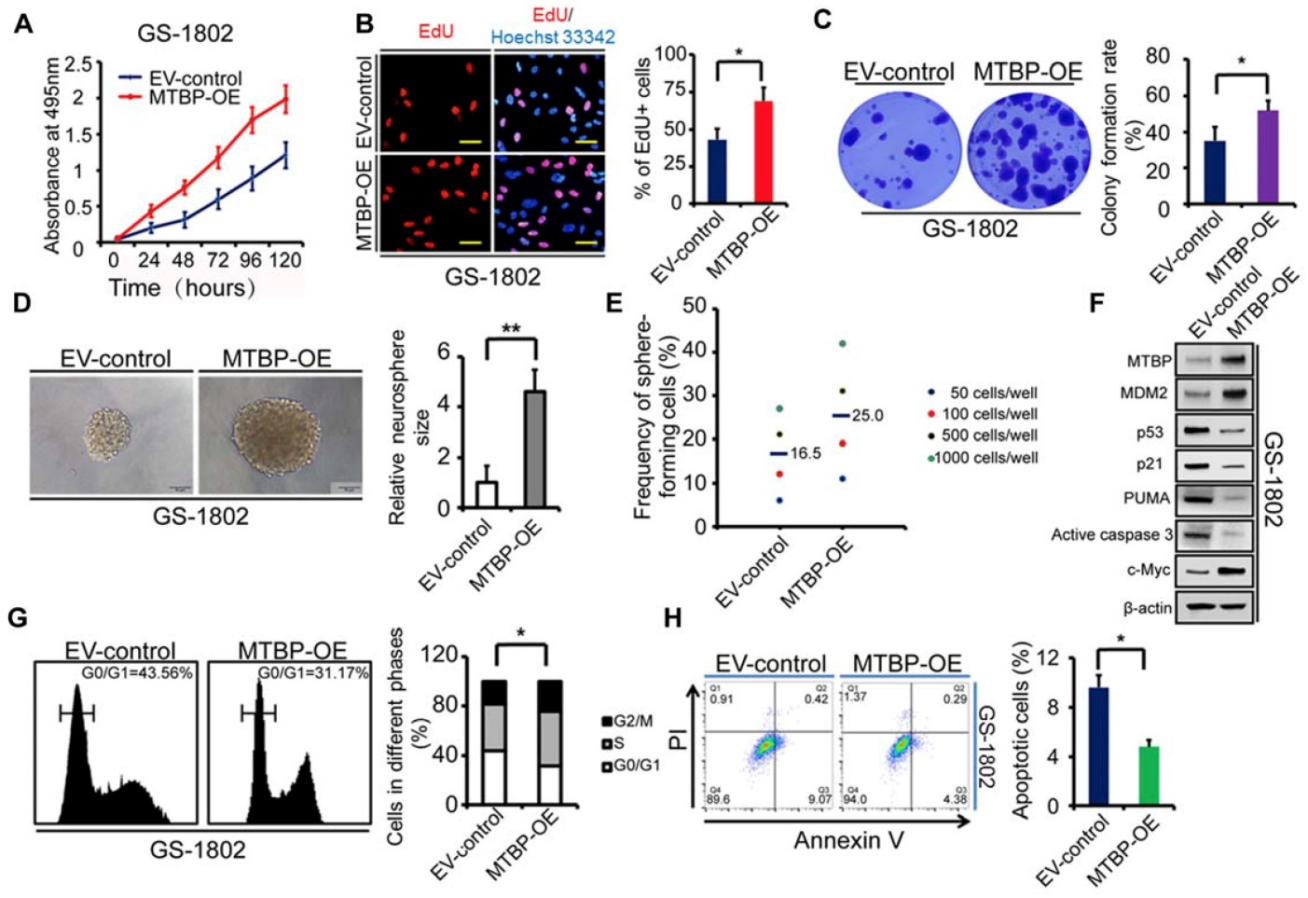
Effect of MTBP overexpression on *in vitro* TP53wt GBM cell growth. A: Effect on GS-1802 cell viability. B: Effect on GS-1802 cell proliferation as determined by EdU incorporation assays. Scale bar: 50 μm. C: Effect on the colony formation of GS-1802 cells as established by soft agar colony assays. D: Representative images of GS-1802 neurospheres transduced with MTBP-overexpressing plasmids or empty vector (left) and quantification of relative neurosphere sizes of indicated GSCs (right). Scale bar, 50 μm. E: Effect on *in vitro* clonogenicity of GS-1802 GSCs as assessed by limiting dilution neurosphere formation assays. F: Western blotting assays of the MTBP, MDM2, p53, p21, PUMA, active caspase3 and c-myc protein levels in GS-1802 cells transfected with MTBP-overexpressing plasmids or empty vector. G: Flow cytometry analysis of GS-1802 cells with MTBP overexpression. H: Annexin V/PI staining and flow cytometry analyses of GS-1802 cells with MTBP overexpression. Results are presented as mean ± SEM of triplicate samples from three independent experiments. *P<0.05, **P < 0.01. OE: overexpression. EV: empty vector.

To further confirm the role of MTBP in TP53wt GBM cells, MTBP was overexpressed in GS-1802 cells that had low basal MTBP levels (Figure 2E). The results show that the upregulation of MTBP remarkably increases viability (Figure 4A; P<0.05), proliferation (Figure 4B; P=0.019), and colony formation (Figure 4C; P=0.035) in TP53wt GS-1802 cells. Furthermore, MTBP overexpression significantly enhances the self-renewal of GSCs (Figure 4D and E; P=0.004). It also leads to the elevation of MDM2 and c-myc protein levels while decreasing those of p53, p21, PUMA, and active caspase3 (Figure 4F), which allows the cells to easily enter the S phase (Figure 4G; P=0.032) and inhibits apoptosis (Figure 4H; P=0.043). In summary, MTBP overexpression suppresses p53-induced apoptosis and promotes growth in TP53wt GBM cells.

### Effect of MTBP on the in vivo tumor growth of TP53wt GSCs

**Figure 5:**
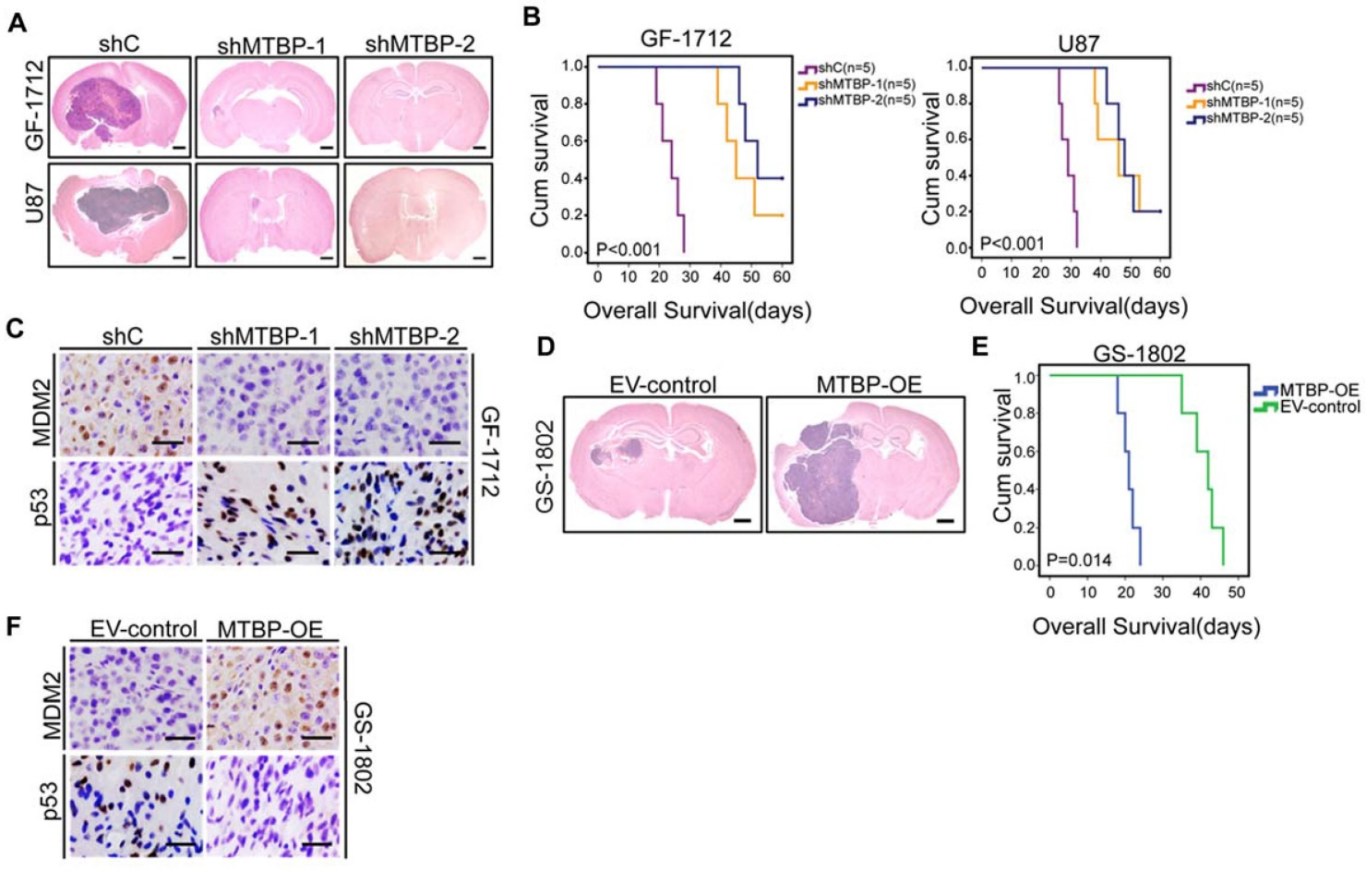
Effect of MTBP on* in vivo* TP53wt GSCs growth. A: Representative HE stained images of mice brain sections after intracranial transplantation of TP53wt GSCs transfected with shC, shMTBP-1, or shMTBP-2. Brains were harvested at 15 days after transplantation. Scale bar: 1 mm. B: Kaplan-Meier survival curves of mice injected with TP53wt GSCs transfected with shC, shMTBP-1, or shMTBP-2. C: Representative IHC images of MDM2 and p53 proteins in intracranial tumors derived from GF-1712 GSCs transfected with shC, shMTBP-1, or shMTBP-2. Scale bar: 25 μm. D: Representative HE stained images of mice brain sections after intracranial transplantation of GS-1802 GSCs transduced with MTBP-overexpression plasmids or empty vector. Brains were harvested at 15 days after transplantation. Scale bar: 1 mm. E: Kaplan-Meier survival curves of mice injected with GS-1802 GSCs transduced with MTBP-overexpression plasmids or empty vector. F: Representative IHC images of MDM2 and p53 proteins in intracranial tumors derived from GS-1802 GSCs transduced with MTBP-overexpression plasmids or empty vector. Scale bar: 25 μm.

Intracranial mouse models were established for the purpose of evaluating the *in vivo* effects of MTBP knockdown and overexpression. The results presented in (Figure 5) indicate that MTBP silencing in GF-1712 and U87 GSCs significantly inhibits intracranial tumor growth (Figure 5A) and prolongs survival time (Figure 5B; P<0.001). Moreover, orthotopic tumors formed by MTBP-silenced GSCs show lower levels of MDM2 and higher levels of p53 compared to control GSCs-induced tumors (Figure 5C). In addition, the overexpression of MTBP in GS-1802 GSCs enhances *in vivo* tumor growth (Figure 5D) and reduces survival time (Figure 5E; P=0.014). Intracranial tumors formed by MTBP-overexpression GSCs exhibit higher levels of MDM2 and lower levels of p53 compared to control GSC-induced tumors (Figure 5F). These results suggest that MTBP promotes* in vivo* tumor growth of TP53wt GSCs possibly via MDM2-dependent modulation of p53.

### Dependence of the MTBP pro-survival effect on the expression of MDM2 and TP53 status

**Figure 6:**
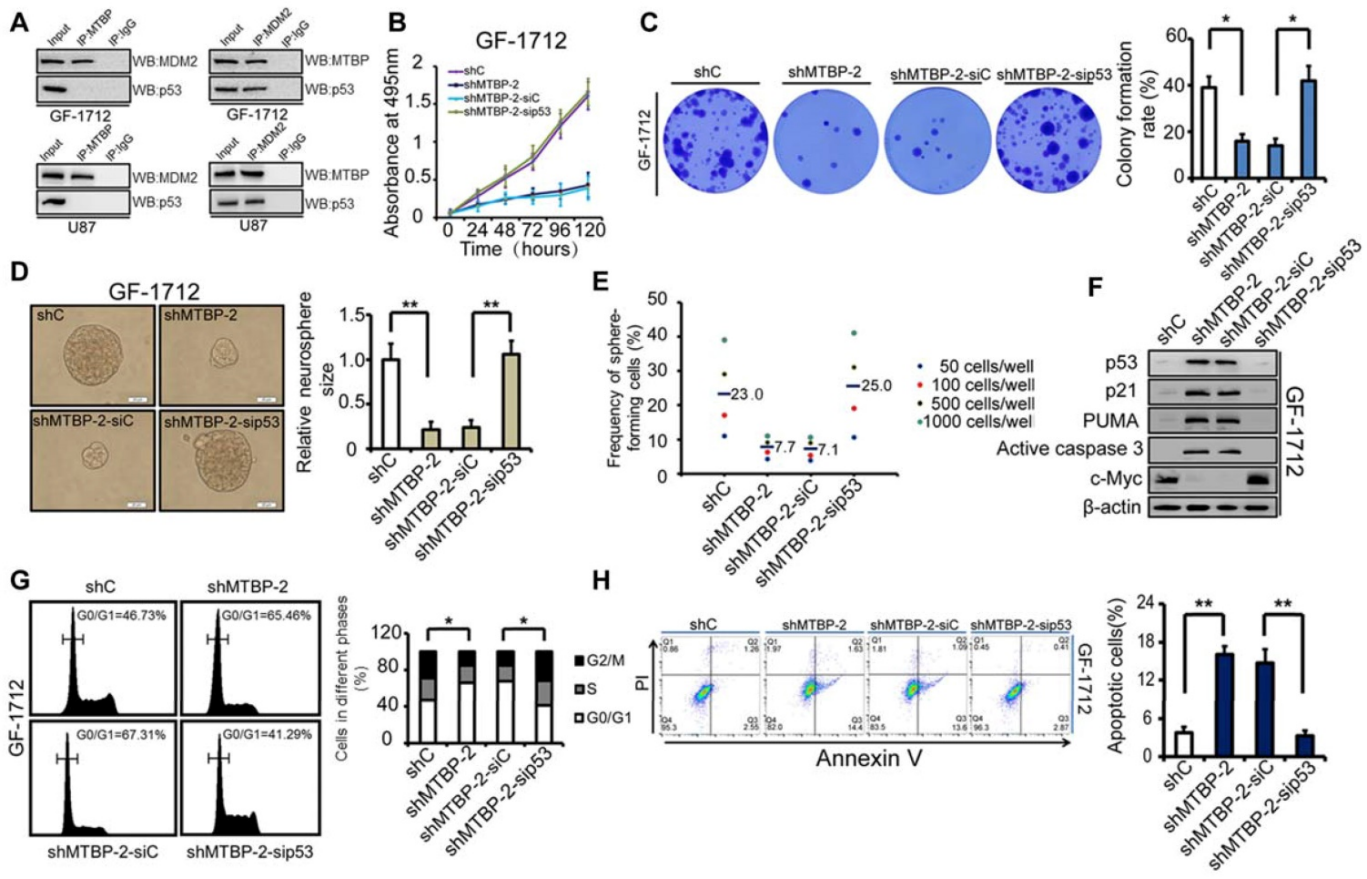
Influence of p53 expression on the pro-survival effect of MTBP in GF-1712 cells. A: MTBP bound to MDM2 but not to p53, whereas MDM2 bound to both MTBP and p53 in GF-1712 and U87 cells as shown by co-immunoprecipitation. B: Effect of p53 silencing on the cell viability of MTBP-knockdown GF-1712 cells. C: Effect of p53 silencing on the colony formation of MTBP-knockdown GF-1712 cells as assessed by soft agar colony assays. D: Representative images of GF-1712 neurospheres transduced with indicated plasmids (left) and quantification of relative neurosphere sizes of indicated GSCs (right). Scale bar: 20 μm. E: Effect of p53 silencing on clonogenicity in MTBP-knockdown GF-1712 cells as determined by limiting dilution neurosphere forming assays. F: Effect of p53 silencing on the expressions of p21, PUMA, active caspase3, and c-myc in MTBP-knockdown GF-1712 cells as established by western blotting analyses. G: Effect of p53 silencing on G0/G1 arrest in MTBP-knockdown GF-1712 cells as shown by flow cytometry. H: Effect of p53 silencing on apoptosis in MTBP-knockdown GF-1712 cells. Results are presented as mean ± SEM of triplicate samples from three independent experiments. *P<0.05, **P < 0.01.

As shown in (Figure 6A), immunoprecipitation demonstrated the binding of MTBP to MDM2 but not to p53, whereas MDM2 bound to both MTBP and p53 in GF-1712 and U87 cells. Therefore, consistent with previous report, these results suggest that MTBP regulates p53 via MDM2 6. The dependence of the MTBP pro-survival effect on the expression of MDM2 was assessed by knocking down MDM2 in MTBP-overexpression GS-1802 cells. It was found that MDM2 silencing markedly inhibits MTBP-induced enhancement of cell viability (Figure S2A; P<0.05), colony formation (Figure S2B; P=0.005), and GSC renewal (Figure S2C and D; P=0.017). Moreover, MDM2 knockdown restores the protein levels of p53, p21, PUMA, and active caspase3 (Figure S2E), as well as induces apoptosis in MTBP-overexpression GS-1802 cells (Figure S2F; P=0.037). It is thus concluded that MTBP promotes the growth of TP53wt GBM cells in an MDM2-dependent manner.

The influence of p53 expression on the pro-survival effect of MTBP in GF-1712 cells was also assessed. The obtained results are illustrated in (Figures 6B-H), which shows that p53 silencing restores cell viability (Figure 6B; P<0.05), colony formation (Figure 6C; P=0.024), and GSC renewal (Figure 6D and E; P=0.001). The results also show that the depletion of p53 decreases the protein levels of p21, PUMA, and active caspase3, increases the expression of c-myc (Figure 6F), relieves G0/G1 arrest (Figure 6G; P=0.035), and inhibits apoptosis (Figure 6H; P=0.001) in MTBP-knockdown GF-1712 cells. These data reveal that MTBP knockdown-induced apoptosis is mediated by increased p53 protein levels. Therefore, in TP53wt GBM cells, the anti-apoptotic effect of MTBP is largely promoted by the depletion of p53 protein. Furthermore, in TP53mut LN229 cells, knocking down MTBP did not significantly influence cell viability, colony formation, GSC renewal, and apoptosis (Figure S3), which confirms that MTBP mainly functions in TP53wt GBM cells via the MDM2-dependent degradation of p53.

### Transcriptional regulation of MTBP by c-myc in TP53wt GBM cells

**Figure 7:**
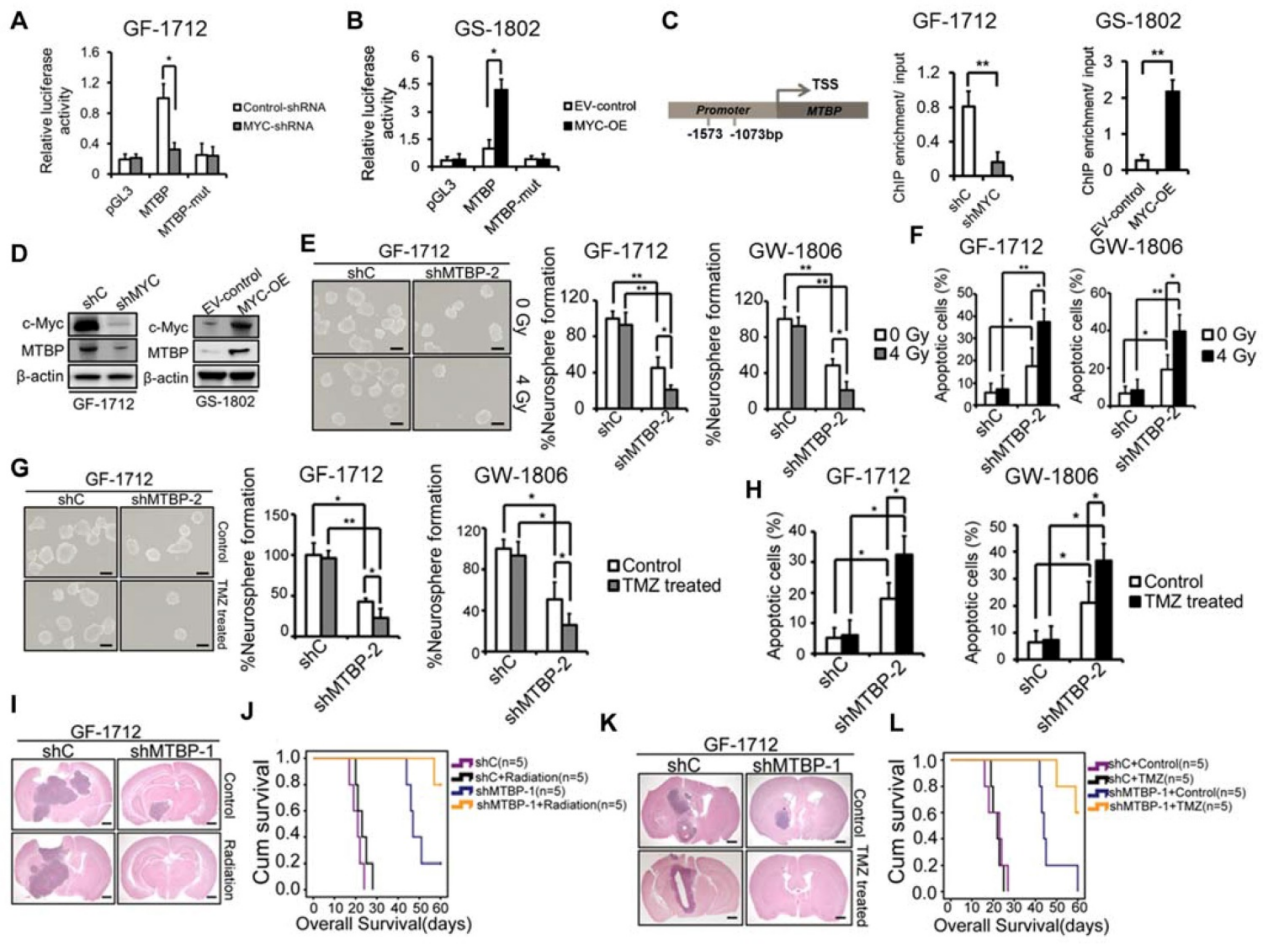
Effect of MTBP silencing on the therapeutic sensitivity of TP53wt GSCs. A and B: Effect of c-myc silencing (A) and overexpression (B) on MTBP promoter activities in GF-1712 and GS-1802 cells respectively as determined by luciferase assays. C: ChIP-qPCR assessment of the binding between c-myc and MTBP promoter. D: Examination of c-myc and MTBP expressions in GF-1712 and GS-1802 cells transduced with indicated plasmids using western blotting analysis. E and F: Effect of 4 Gy radiation exposure on the neurosphere formation rate (E) and the apoptotic rate (F) of TP53wt GF-1712 and GW1806 GSCs previously transduced with either control or shMTBP-2 containing lentivirus. Scale bar: 100 µm. G-H: Effect of 100 μM TMZ treatment on the neurosphere formation rate (G) and the apoptotic rate (H) of GF-1712 and GW1806 GSCs transduced with either control or shMTBP-2 containing lentivirus. Scale bar: 100 µm. I-J: The combined effect of MTBP knockdown and genotoxic treatment in mouse xenograft tumors. Brains were harvested at 20 days after transplantation. Scale bar: 1 mm. Kaplan-Meier survival curves of mice intracranially transplanted GF-1712 GSCs with shC or shMTBP-1 in response to radiation (J; Log-rank P-value: shC *vs.* shC + Radiation: 0.169, shC *vs.* shMTBP-1 : 0.002, shC + Radiation *vs.* shMTBP-1 + Radiation: 0.002, shMTBP-1 *vs.* shMTBP-1 + Radiation: 0.032) or TMZ treatment (L; Log-rank P-value: shC + Control *vs.* shC + TMZ: 0.668; shC + Control *vs.* shMTBP-1 + Control : 0.002; shC + TMZ *vs.* shMTBP-1 + TMZ: 0.001; shMTBP-1 + Control* vs.* shMTBP-1 + TMZ: 0.02). Results are presented as mean ± SEM of triplicate samples from three independent experiments. *P < 0.05, **P < 0.01.

A tumorigenesis-promoting interaction between MTBP and c-myc was identified in a previous study 9. Therefore, in this study, the possibility of transcriptional regulation of MTBP by c-myc was examined. Luciferase reporter assays were conducted such that the MTBP promoter, with and without mutation in the c-myc-binding site, was cloned upstream of the luciferase. The results presented in (Figure 7) show that c-myc silencing reduces luciferase activity in GF-1712 cells with wildtype promoters (Figure 7A; P=0.025), while c-myc overexpression remarkably enhances the MTBP-promoter activity in GS-1802 cells (Figure 7B; P=0.016). Moreover, mutation in the c-myc-binding site significantly diminishes luciferase activity compared to the wildtype promoter (Figures. 7A and B). ChIP assay results confirm the binding of c-myc to the MTBP promoter. Silencing of c-myc significantly inhibits this binding in GF-1712 cells, whereas the overexpression of the protein markedly increases it in GS-1802 cells (Figure 7C; P<0.01). Meanwhile, western blotting analysis shows that c-myc silencing decreases MTBP expression, while overexpression of the protein increases it (Figure 7D).

### Effect of MTBP silencing on the therapeutic sensitivity of TP53wt GSCs

Activation of p53 has been reported to sensitize GBM to radio-chemotherapy 20, 21. In this study, we further examined whether MTBP silencing could influence the therapeutic sensitivity of TP53wt GSCs that are responsible for treatment resistance. The results show that both, GF-1712 and GW-1806 GSCs, carry the unmethylated MGMT promoter (Table S1) and are resistant to radiation and TMZ (Figures. 7E-H). MTBP silencing significantly increases the sensitivity of TP53wt GSCs to radiation, which promotes neurosphere formation inhibition (Figure 7E; P<0.05) and apoptotic cell accumulation (Figure 7F; P<0.05) upon exposure to radiation. The same enhanced sensitivity to genotoxic treatment was observed in TP53wt GSCs treated with TMZ; i.e., the loss of MTBP promotes TMZ-induced inhibition of neurosphere formation (Figure 7G; P<0.05) and increases TMZ-induced apoptosis (Figure 7H; P<0.05). Next, we investigated the combined effect of MTBP knockdown and genotoxic treatment in mouse xenograft tumors using TP53wt GF-1712 GSCs. Consistent with our* in vitro* findings, radiation or TMZ treatment alone did not significantly affect the *in vivo* growth of GF-1712 GSCs, whereas silencing MTBP prolonged the survival of the mice. Moreover, combination of MTBP inhibition with radiation or TMZ treatment showed a further reduction in tumor growth, and significantly prolonged mouse survival (Figure 7I-L; Log-rank P<0.05). Thus, targeting MTBP can affect the biological response to genotoxic treatment.

## Discussion

GBM is one of the most lethal human malignancies, with a median patient survival duration of about 15 months, due to its resistance to radiation and other genotoxic treatments 1. A subpopulation of tumor cells with potent tumorigenic abilities, known as glioma stem cells (GSCs), contributes significantly to the therapy resistance and regrowth of this tumor, even after aggressive treatment 2, 22. However, the molecular mechanisms underlying tumor resistance to radio-chemotherapy remain elusive.

The tumor suppressor gene p53 may prevent tumor development by induction of apoptotic death in nascent neoplastic cells 23. P53 also opposes the self-renewal of normal and cancer stem cells 3, 24. In addition, p53 plays a major role in the response of malignant cells to anticancer therapies that cause DNA damage 25. Consistent with our results, previous studies show that this gene directly upregulates the expressions of p21, PUMA, and active caspase3 and downregulates the expression of c-myc, thereby controlling cell cycle arrest, cell senescence, and cell death 23. To facilitate malignant progression, TP53 is mutated in about 50% of human cancers 26. However, more than two-thirds of human GBMs have a wildtype p53 gene, which, in theory, should retain its function 27. Paradoxically, GBM is notoriously resistant to the currently available radio-chemotherapy treatments. The attenuation of p53 functions in GBM patients has been attributed to post-translational regulation of the p53 protein, whose levels are tightly controlled by the E3 ubiquitin ligase MDM2 through proteasomal degradation 28. Elevated levels of MDM2 protein impair wildtype p53 function and accelerate tumor growth in various human cancers, including leukemia, sarcoma, breast cancer, melanoma, and GBM 7, 29-32. Nevertheless, MDM2 also transfers ubiquitin to itself, resulting in self-destruction 6.

**Figure 8:**
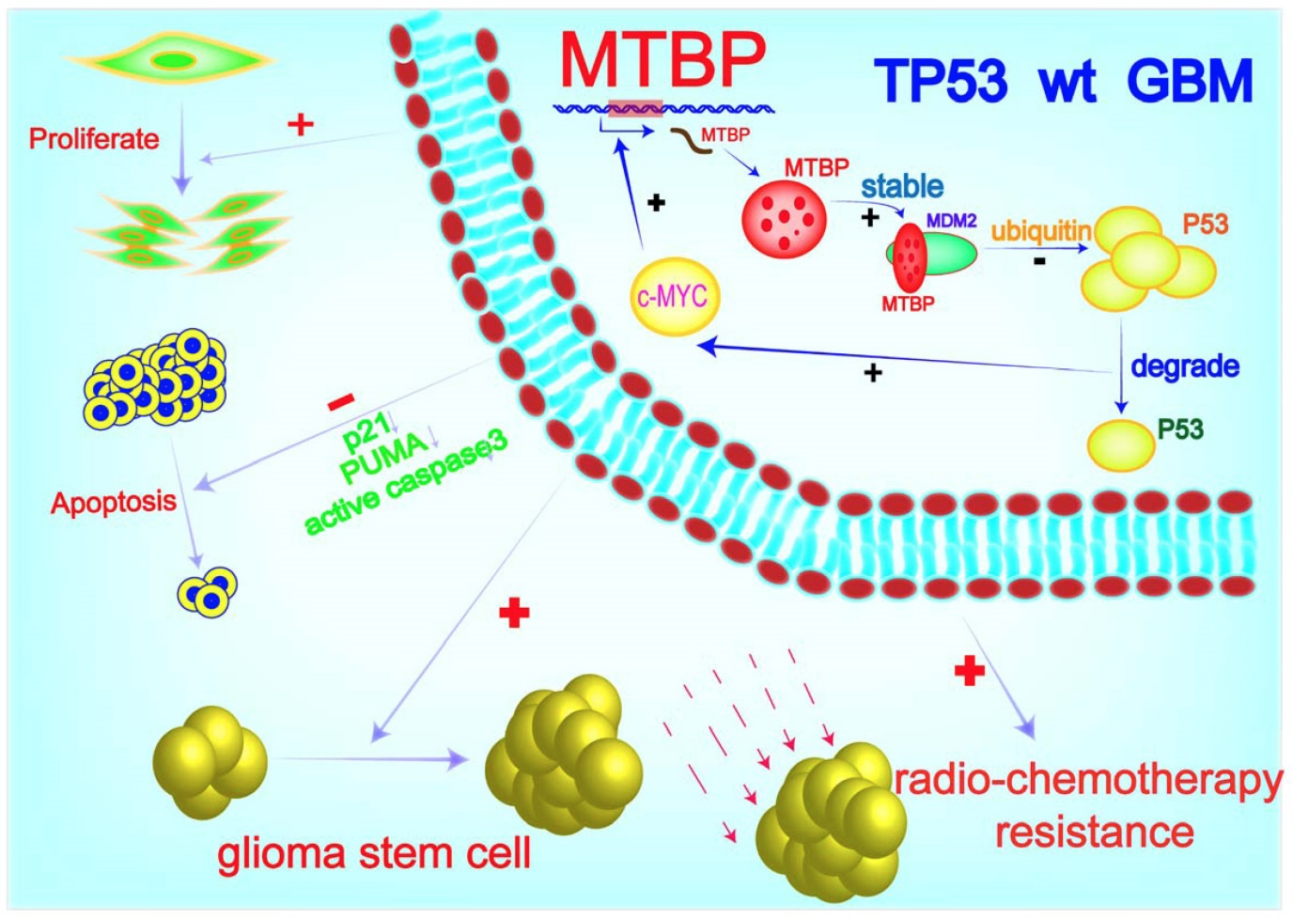
A working model of MTBP-regulated cell survival and treatment resistance in TP53wt GBMs. MTBP promotes the survival and treatment resistance of TP53wt GBM cells through MDM2-dependent post-translational modification of p53.

MTBP possesses the ability to differentially regulate the ubiquitin ligase activity of MDM2 toward itself and p53 6. Consistently, we found that MTBP protein expression is positively and negatively correlated with MDM2 and p53 protein levels in TP53wt GBMs, respectively. Through MDM2 stabilization and p53 degradation, MTBP inhibits the expression of the p53 effectors of apoptosis and cell cycle arrest/cell senescence, upregulates the expression of c-myc oncoprotein, and promotes tumor growth and treatment resistance. Although the role of MTBP in the invasion of tumor cells is still controversial 33, 34, its pro-proliferative effect via interaction with c-myc has been reported in several human malignancies, including breast cancers 35, B-cell lymphomas 36 and lung cancers 9. In B-cell lymphoma, MTBP expression is induced by c-myc, suggesting that MTBP might be a transcriptional target of c-myc 36. In this study, we show that c-myc positively regulates the transcription of MTBP by binding to its promoter. Our results indicate that in TP53wt GBM cells, MTBP promotes the expression of c-myc through MDM2-mediated degradation of p53 protein; meanwhile, c-myc further upregulates the expression of MTBP at the transcription level. This MTBP-MYC positive regulatory loop may play an important role in the malignant growth and therapy resistance of TP53wt GBMs. Furthermore, we show that MTBP knockdown restores the function of p53, thereby inhibiting tumorigenicity and increasing the sensitivity of GSCs to radiation and TMZ treatment. Therefore, targeting MTBP may be potentially utilized in TP53wt GBM therapy (Figure 8).

## Conclusion

In this study, we identified a novel mechanism of p53 regulation in TP53wt GBMs, which may provide a therapeutic advantage of reducing tumor recurrence. MTBP enhances cell survival and treatment resistance through MDM2-mediated degradation of p53 protein. Targeting MTBP might be of potential use for the treatment of TP53wt GBM patients.

## Supplementary Material

Supplementary figures and table.
