# Supplementary material for: MTBP regulates cell survival and therapeutic sensitivity in TP53 wildtype glioblastomas
Source: Theranostics. 2019 Aug 14;9(20):6019–30. doi: 10.7150/thno.35747 (PMC6735364; doi:10.7150/thno.35747)
Supplement: Supplementary file 1 — Supplementary figures and table. [file thnov09p6019s1.pdf]

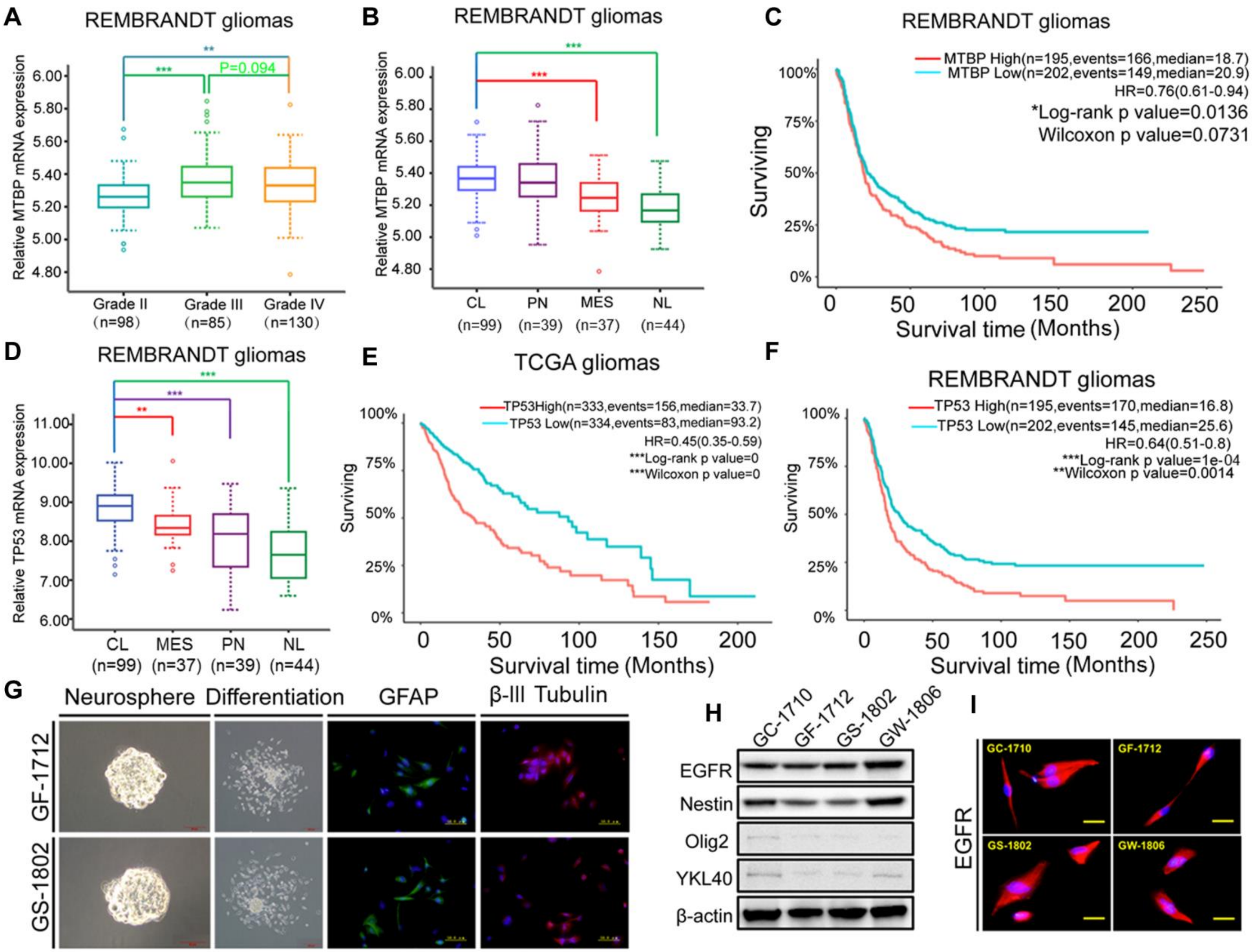

Supplementary Figure 1

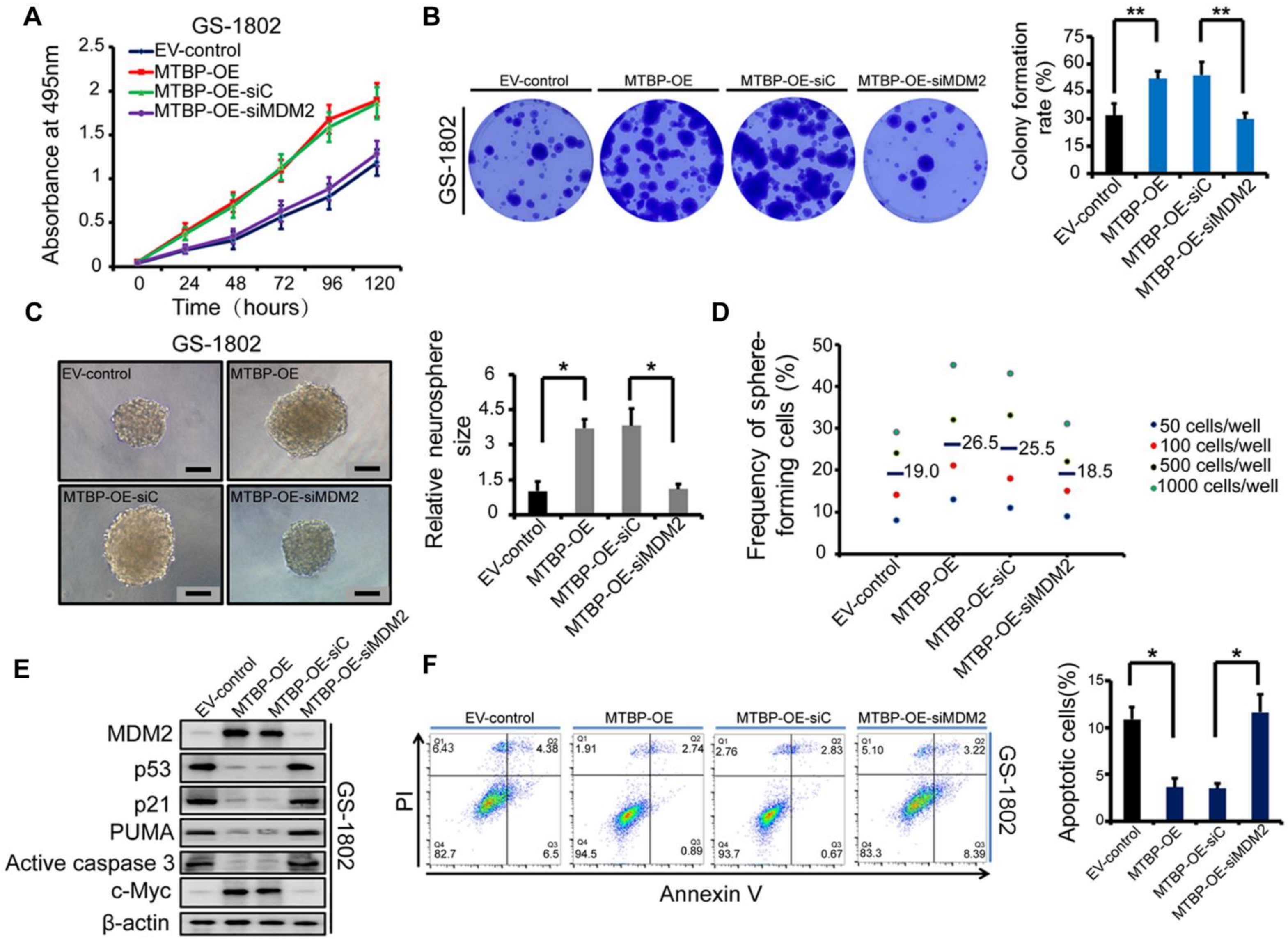

Supplementary Figure 2

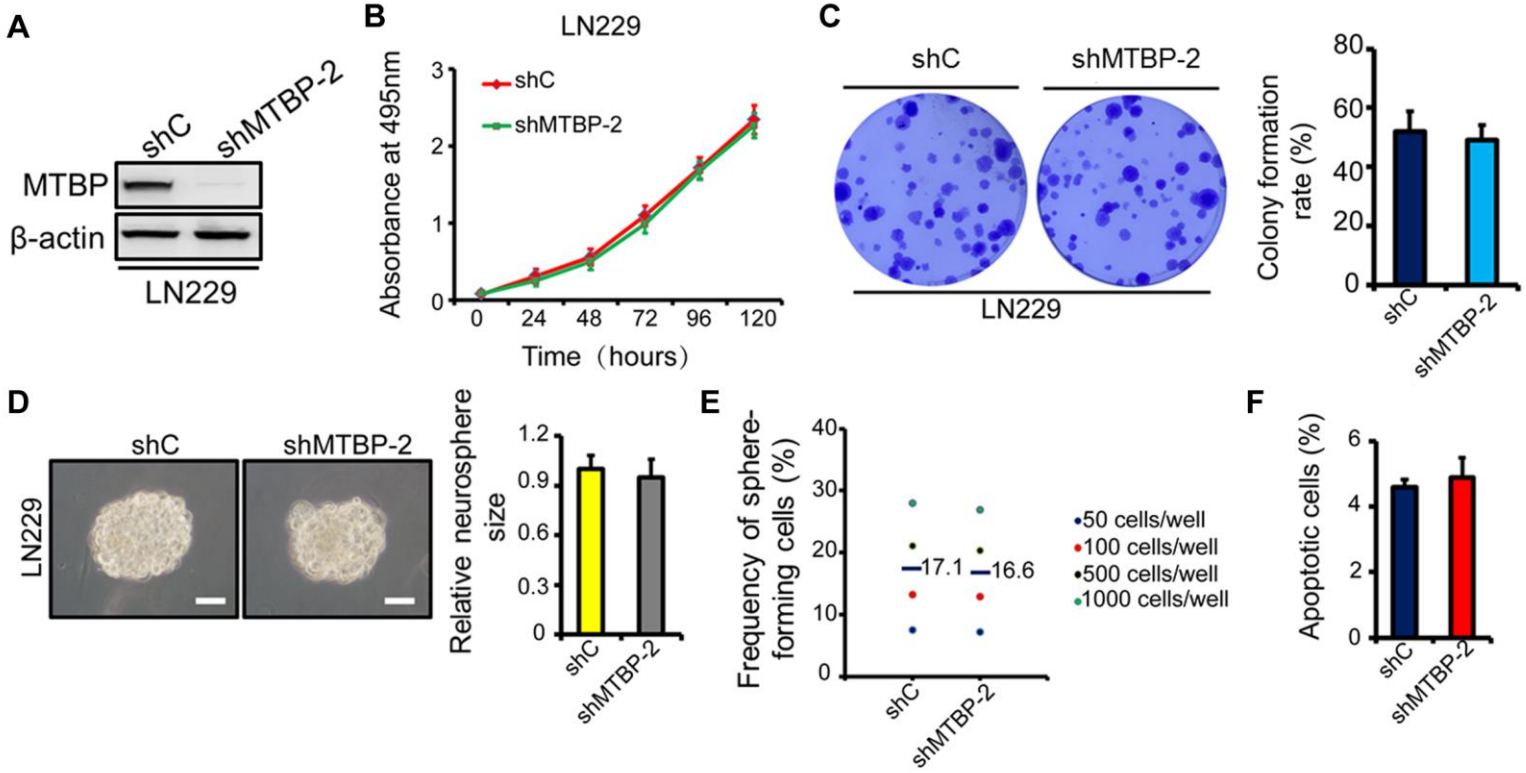

Supplementary Figure 3

Supplementary Figure Legends

Supplementary Figure 1. Influence of pathological and molecular glioma features on MTBP and TP53 expressions. A: Influence of REMBRANDT glioma histopathologic grades on MTBP mRNA expression. B: Influence of REMBRANDT glioma molecular subtypes on MTBP mRNA expression. C: Prognostic significance of MTBP in REMBRANDT gliomas. D: Influence of REMBRANDT glioma molecular subtypes on TP53 mRNA expression. E and F: The prognostic role of TP53 mRNA expression in TCGA (E) and REMBRANDT (F) gliomas. G: Multi-lineage differentiation capacity of GSCs. H and I: The expression of classical markers (EGFR and Nestin), proneural marker (Olig2) and mesenchymal marker (YKL40) was examined in GC-1710, GF-1712, GS-1802, and GW-1806 cells using western blot (H) or immunofluorescence (I).***P < 0.001, **P < 0.01. Classical, CL; neural, NL; proneural, PN; and mesenchymal, MES.

Supplementary Figure 2. Dependence of the MTBP pro-survival effect on the expression of MDM2 in TP53wt GBM cells. A: Effect of MDM2 silencing on MTBP-induced proliferation of GS-1802 cells. B: Effect of MDM2 knockdown on MTBP-induced colony formation in GS-1802 cells as determined by soft agar colony assays. C: Representative images of GS-1802 neurospheres transduced with indicated plasmids (left) and quantification of relative neurosphere sizes of indicated GSCs (right). Scale bar: 50 μm. D: Effect of MDM2 silencing on MTBP-induced *in vitro* clonogenicity of GS-1802 GSCs. E: Effect of MDM2 silencing on the MTBP-induced downregulation of p53, p21, PUMA, and active caspase3 and upregulation of c-myc in GS-1802 cells as determined by western blot analyses. F: Effect of MDM2 knockdown on the apoptosis of MTBP-overexpressing GS-1802 cells. Results are presented as mean ± SEM of triplicate samples from three independent experiments. *P < 0.05, **P < 0.01.

Supplementary Figure 3. Effect of MTBP silencing on the growth of TP53mut GBM cells. A: Western blotting analysis of TP53mut LN229 cells transduced with shRNA targeting MTBP (shMTBP-2) or a control shRNA (shC). B: Effect of MTBP knockdown by shRNA on cell viability in LN229 cells. C: Effect of MTBP knockdown on colony formation in LN229 cells, as assessed by soft agar colony assays. D: Representative images of LN229 neurospheres transduced with shMTBP-2 or shC (left) and quantification of relative neurosphere sizes of indicated GSCs (right). Scale bar: 50 μm. E: Effect of MTBP silencing on the clonogenicity of LN229 GSCs, as determined by limiting dilution neurosphere formation assays. F: Effect of MTBP silencing on the apoptosis of LN229 cells. Results are presented as mean ± SEM of triplicate samples from three independent experiments.

Supplementary Table 1. Clinical information of the primary glioma cells

	GC-1710	GF-1712	GS-1802	GW-1806
**Gender**	Male	Female	Male	Female
**Age**	68 years old	72 years old	67 years old	48 years old
**Location**	Right temporal lobe	Right occipital lobe	Left temporal lobe	Left frontal lobe
**Pathological diagnosis**	Glioblastoma	Glioblastoma	Glioblastoma	Glioblastoma
**WHO grade**	IV	IV	IV	IV
**Ki-67**	15% (+)	30% (+)	30% (+)	40% (+)
**TP53**	Wildtype	Wildtype	Wildtype	Wildtype
**1p/19q**	Non-codeletion	Non-codeletion	Non-codeletion	Non-codeletion
**IDH1**	Wildtype	Wildtype	Wildtype	Wildtype
**IDH2**	Wildtype	Wildtype	Wildtype	Wildtype
**TERT**	Mutant	Mutant	Mutant	Mutant
**MGMT promoter**	methylated	unmethylated	unmethylated	unmethylated
